# A synthesis of European seahorse taxonomy, population structure, and habitat use as a basis for assessment, monitoring and conservation

**DOI:** 10.1007/s00227-017-3274-y

**Published:** 2017-12-05

**Authors:** Lucy C. Woodall, Francisco Otero-Ferrer, Miguel Correia, Janelle M. R. Curtis, Neil Garrick-Maidment, Paul W. Shaw, Heather J. Koldewey

**Affiliations:** 1 Department of Zoology, University of Oxford, Oxford, UK; 2 Project Seahorse, Zoological Society of London, Regent’s Park, London, UK; 3 Grupo en Biodiversidad y Conservación, IU-ECOAQUA, Universidad de Las Palmas de Gran Canaria, Crta. Taliarte s/n, 35214 Telde, Spain; 4 CCMar, Universidade do Algarve, F. C. T., Edificio 7, Campus de Gambelas, 8005-139 Faro, Portugal; 5 Pacific Biological Station, Fisheries and Oceans Canada, 3190 Hammond Bay Road, Nanaimo, BC Canada; 6 The Seahorse Trust, 36 Greatwood Terrace, Topsham, Devon UK; 7 Institute of Biological, Environmental and Rural Sciences (IBERS), Aberystwyth University, Aberystwyth, UK; 8 Centre for Ecology and Conservation, University of Exeter, Penryn, UK

## Abstract

Accurate taxonomy, population demography, and habitat descriptors inform species threat assessments and the design of effective conservation measures. Here we combine published studies with new genetic, morphological and habitat data that were collected from seahorse populations located along the European and North African coastlines to help inform management decisions for European seahorses. This study confirms the presence of only two native seahorse species (*Hippocampus guttulatus* and *H. hippocampus*) across Europe, with sporadic occurrence of non-native seahorse species in European waters. For the two native species, our findings demonstrate that highly variable morphological characteristics, such as size and presence or number of cirri, are unreliable for distinguishing species. Both species exhibit sex dimorphism with females being significantly larger. Across its range, *H. guttulatus* were larger and found at higher densities in cooler waters, and individuals in the Black Sea were significantly smaller than in other populations. *H. hippocampus* were significantly larger in Senegal. *Hippocampus guttulatus* tends to have higher density populations than *H. hippocampus* when they occur sympatrically. Although these species are often associated with seagrass beds, data show both species inhabit a wide variety of shallow habitats and use a mixture of holdfasts. We suggest an international mosaic of protected areas focused on multiple habitat types as the first step to successful assessment, monitoring and conservation management of these Data Deficient species.

**Electronic supplementary material:**

The online version of this article (10.1007/s00227-017-3274-y) contains supplementary material, which is available to authorized users.

## Introduction

The paucity of species-specific data is among the many challenges to designing effective marine conservation measures that are resilient to the enduring threats of climate change, coastal development, over-fishing, by-catch effects and invasive species (Klein et al. 2013; Selig et al. 2014). These challenges are further compounded when the taxonomy of species is uncertain. Knowing which species occur and understanding their life-history, ecology, and behaviour is increasingly important to ensure effective and robust conservation and management (Perry et al. 2005; Lavergne et al. 2010; Dawson et al. 2011).

The cryptic nature of seahorses (genus *Hippocampus*) has led to significant confusion regarding their taxonomy and ecology, which poses challenges to managing the activities that threaten these fishes. The most recent and comprehensive taxonomic review suggests there are two native species of seahorse in European waters, *H. guttulatus* and *H. hippocampus* (Lourie et al. 2016), but considerable intraspecific variability in morphology within this genus (Lourie et al. 1999b; Otero-Ferrer et al. 2017) has led to much confusion regarding their taxonomy, and the taxonomy and nomenclature of these species is not stable. Authors previously suggested many additional species within this geographic range, based on small morphometric differences (Kuiter 2009). For instance a study by Vasil’Eva (2007), which has not been adopted (Eschmeyer and Fricke 2016), attempted to change the names of these species, while another author suggested additional species were present based on photographs (Kuiter 2009). There is ongoing discussion as to whether *H. ramulosus* is a simple synonym of *H. guttulatus*, and whether the regional morphological differences observed across the seahorse populations in the region are indicative of different species (Kuiter 2009). Taxonomic controversy involving splitting and lumping of species is common throughout the Syngnathidae family, due to limited discriminating morphological characteristics between species and the ability within the family to change colour and cirri (filamentous skin appendages) (Curtis 2006). As most ecological studies of seahorses in Europe have used the nomenclature of *H. hippocampus* and *H. guttulatus* to define their focal species (e.g. Curtis and Vincent 2005; Kitsos et al. 2008; Ben Amor et al. 2011; Caldwell and Vincent 2012; Filiz and Taskavak 2012; Gristina et al. 2015), there is some consensus for a conservative view of seahorse taxonomy. Some reports also suggest range extensions into European waters by non-native species: *H. algiricus* presence in the Canary Islands (Otero-Ferrer et al. 2015b, 2017), the Lessepsian migrant *H. fuscus* in the eastern Mediterranean (Golani and Fine 2002), and occasional rare migrants (e.g. *H. erectus*, Woodall et al. 2009). Therefore genetic data are particularly useful to clarify taxonomy and complement morphological data (Padial et al. 2010).

The two European seahorses *H. guttulatus* and *H. hippocampus* are the currently recognised names used in the IUCN Red List of Threatened Species (2012) and both are currently assessed as Data Deficient (Woodall 2012a, b). Both species have a large geographic range extending across most of Europe and North Africa including the Atlantic Ocean, Mediterranean and Black Seas (Lourie et al. 1999b; Otero-Ferrer et al. 2017). Neither species is thought to be currently targeted by fisheries throughout most of their geographic range, but there is trade in west Africa of *H. hippocampus* (Cisneros-Montemayor et al. 2016) and a new and increasing fishery for *H. guttulatus* in the Ria Formosa in Portugal (M. Correia, pers. obs.). Both species are also susceptible to anthropogenic activities and habitat loss (Curtis et al. 2007). Ecological data on seahorses are scarce due to their apparent patchy distribution and low density, as well as their cryptic nature (Foster and Vincent 2004). These features make them particularly difficult to survey, assess and monitor the status of their populations, either for scientific research or commercial development projects, such as environmental impact assessments prior to construction work.

To date, a range-wide ecological assessment has been conducted for just one seahorse species (*H. capensis*), which is confined to three estuaries in South Africa (Lockyear et al. 2006). For European seahorses, research has generally been limited to small focal sites (e.g. Curtis and Vincent 2006; Gristina et al. 2015) or collection of qualitative data (e.g. Filiz and Taskavak 2012). However, a very large sighting dataset has been collected for UK and Ireland (N. Garrick-Maidment pers. comm.). Comparisons of population structure among studies is also challenging because seahorse length can be measured by standard length (*L*_S_), total length (*L*_T_) or height (Lourie et al. 1999a) and previous studies have used all of these (e.g. Verdiell-Cubedo et al. 2008; Nadeau et al. 2009; Caldwell and Vincent 2012; Vieira et al. 2014).

Focusing on the taxonomy, biology and life history of European seahorses, we use published and unpublished sources of genetic, demographic and environmental data to investigate the following objectives:

1. Use genetic markers to confirm the number of seahorse species present in Europe
2. Test for differences in population structure and behaviour throughout the range
3. Test for the correlation of population structure and morphology with environmental variables

This information will help to advance our ability to effectively manage *Hippocampus* spp. within Europe, by providing detailed information that can help determine appropriate protection and mitigation interventions as well as the accurate assessment of seahorse populations.

## Materials and methods

### Geographic extent, literature sources and standardization

The geographic extent of this review covers seahorse populations from the Northeast Atlantic Ocean, including the Macaronesian islands, and the Mediterranean and Black Seas. In total, data from 13 countries and 37 different sites are reviewed. These data cover the known geographic range of *H. guttulatus* and *H. hippocampus* (Lourie et al. 1999b), however most individual studies generally focused on sites in Portugal and the eastern Mediterranean due to these being identified as having relatively high seahorse abundance that led to longer term studies. Data used in this review were from a wide range of sources, including sources known to authors or found using a combination of the following search terms ‘Seahorse, *Hippocampu*s, *guttulatus*, *ramulosus*, Mediterranean, Atlantic, Black Sea, short snouted, long snouted, Hippocampe, Caballito de Mar, cavalos marinhos’ in search engines Google Scholar and Web of Science. Sources include new data and published literature comprising peer-reviewed papers, theses, books and grey literature such as conference posters and reports. Due to the diversity of the methods employed by these studies, we were not able to use all data from all studies in comparisons among sites. However, all density measures were standardised to ind. m^−2^ and seahorse length measurements were compiled as standard length (*L*_S_) (Curtis and Vincent 2006), height (Foster and Vincent 2004) or total length (*L*_T_) (Verdiell-Cubedo et al. 2008).

### New sample acquisition and genetic analysis

**Fig. 1:**
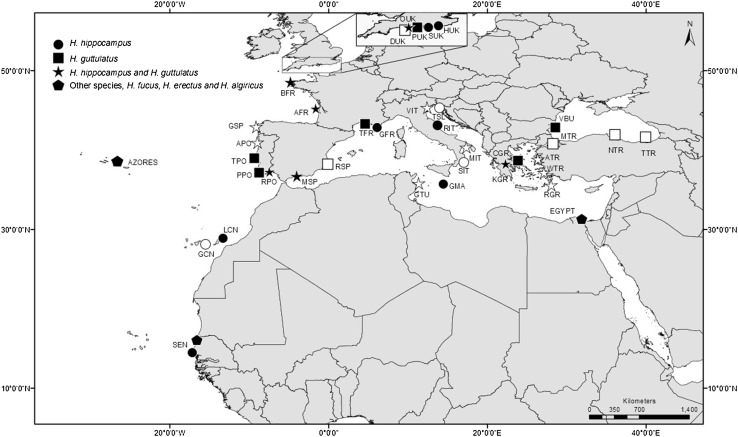
Locations of seahorse tissue collection, population demography and environmental data, including site codes. Filled shapes are sites with new data and open shapes are sites with published data

Most specimen tissue was collected during sampling dives. Further specimens or tissue and associated data were also donated by fishers, public aquariums and academics, and were used when source location was known. In total, seahorse tissue was obtained from specimens from 18 sites around Europe and North Africa (Fig. 1). Authors directly sampled tissues from 14 sites, while tissue from the remaining four locations was donated by other researchers. The mitochondrial DNA cytochrome b gene (cytb) and Control Region (CR) were amplified from specimens using methods given in Woodall et al. (2011, 2015). All DNA sequences were deposited in Genbank (Table S1). Cytb is most routinely sequenced in seahorses, and thus provided an opportunity to include the greatest number of species. The cytb sequences were combined with seahorse reference sequences (Casey et al. 2004; Teske et al. 2007a, b), and aligned using ClustalW (Larkin et al. 2007) implemented in Geneious v6.1.7. The pipefish *Syngnathus temminckii* was used as an outgroup. A phylogenetic tree, to group similar haplotypes, was created in Mr. Bayes (Huelsenbeck and Ronquist 2001) implemented in Geneious after using Find model (Posada and Crandall 2001) to determine the most suitable nucleotide substitution model (GTR + γ) for this dataset.

### Environmental and demographic data collection

Environmental and demographic data were collected opportunistically during SCUBA dives, and morphological data from donated specimens as detailed below. Ideally, a stratified random or systematic sampling regime would be used to capture the full range of diversity in genetic and demographic structure and to identify environmental correlates. However, this was not feasible at the geographic scale of interest because so little information is known of seahorse distributions, which are generally patchy and low-density (Curtis and Vincent 2005). Instead local knowledge of seahorse occurrences was used to identify suitable and accessible survey locations. All seahorses encountered were identified in the field as either *H. guttulatus* or *H. hippocampus*, using morphological characteristics that have proved to be robust such as head shape and head:snout ratio (Lourie et al. 1999b; Curtis 2006). Photographs of specimens were taken when possible and representative images displayed in Fig. S1. Water temperature was extracted from http://www.seatemperature.org (1st July 2015) and was used for all sites where published studies were conducted.

#### Survey data collected by divers

At 14 of the 37 sites, diving methods were employed to sample seahorse populations in two ways: (a) to collect tissue samples for genetic studies (collection dives—see Woodall et al. 2011); or (b) to carry out rapid population assessments (transect dives—adapted from Curtis and Vincent 2005; Woodall et al. 2015). All dives were conducted during the main breeding season for the seahorses (May–October, Curtis and Vincent 2006). During both dive types (collection and transect), once individual seahorses were located, their holdfast (seagrass species, algae species, artificial structures, sand, shells, sessile invertebrates), depth and macro habitat (seagrass, macroalgae, sessile invertebrates, sponge, sand/mud, stones/pebbles, rocks, cliff or artificial structures) were recorded, as well as species, sex, maturity (size when brood pouch is mature in males of that population; Curtis and Vincent 2006; Curtis et al. 2017), presence or absence of cirri (Curtis 2006) and straight trunk length (*L*_Tr_) which was later converted to standard length (*L*_S_) (Curtis and Vincent 2006). Water clarity was assessed during each dive by estimating the horizontal visible distance (meters). The mean temperature at each site was calculated and recorded using a dive computer (Mosquito, Suunto).

- Collection dives
  Between two and five (site dependant) divers searched the benthic substratum for seahorses using a random search pattern. The total time spent searching (diver hours) and number of individual seahorses located were recorded, but using this search pattern it was not possible to record the area of benthos searched. Search effort was measured by time and the number of searching divers, so the relative abundance of seahorses (seahorse per diver hour) could be reported (Schmitt and Sullivan 1996).
- Transect dives
  A random position within the general search area was assigned as the starting point. This point was defined either by its GPS position or by its bearing and distance from a known structure (e.g. pier or rocky feature). From the starting position, a 30 m tape was laid out by one diver in a random direction while the other diver recorded the number of seahorses by species, sex and holdfast within a 2 m corridor belt transect centred along the tape length. Returning along the transect, both divers assessed the habitat by determining the dominant habitat type, which was defined by the broad categories given above. The divers also determined the percentage of cover of each habitat type within three randomly positioned 1 m^2^ quadrats. This process was repeated so that a total of four transects were surveyed per site. At one site in Greece (KGR—Fig. 1), the transects originated from a start line running at right angles to the slope. These transects were positioned to run parallel to the slope contours, at randomly assigned distances along the starting line. The deepest transect was surveyed first and the shallowest last. This method was necessary at KGR as it had a rapidly sloping benthic profile, which was absent from other sites.

#### Commercial trade and fishing data

Fisher data were collected from two locations in the UK. The seahorses were accidentally captured in gill nets and crab pots by local fishers who were targeting *Solea solea* and *Cancer pagurus*. Undamaged seahorses were returned to the water and injured ones were donated to local public aquariums. Environmental (habitat and depth) and seahorse-specific data (species and number) were recorded on the fishing boat and at the aquariums. Seahorses were donated by researchers from three locations in France, Portugal and Italy (AFR, TPO and RIT Table 1, Fig. 1). Seahorses from site AFR were collected during experimental trawls that were used to survey fish diversity in the bay. These seahorses were returned to their collection site following *L*_Tr_ measurement and photographing. Sites TPO and RIT were fished using beach seine nets by aquarium staff for specimen provision to local public aquariums. Specimens were measured and photographed by aquarium staff. Specimens from Senegal (SEN) were donated by Project Seahorse. Most of these Senegalese samples were obtained from a traditional medicine market (Hong Kong) by representatives of a Project Seahorse/TRAFFIC partnership, but twelve were obtained by K. West directly from Senegal traders (West 2012).

**Table 1:** Sample sites of seahorses *Hippocampus guttulatus* (G) and *H. hippocampus* (H), sampling method [Fishing Method (Net, Pot, Trawl, Trammel or Dredge), Type of dive (Collection, Survey or Transect), Social (Donations, Interviews or Trade)], and environmental parameters (depth, visibility, temperature, habitat and main seahorse holdfast), (a) new data (b) published data

(a) New data									
Site	Country	Location type	Species	Sampling method	Depth range (m)	Visibility (m)	Temperature (°C)	Holdfast	Habitat
HUK	UK	Coast	H	Fisher–Nets	≈ 55	n/a	n/a	*Plocamium* spp.	Sand and macroalgae
SUK	UK	Coast	H	Fisher–Pots	≈ 25	n/a	n/a	n/a	Mussel bed
BFR	France	Coastal	H, G	Collection dive	2–6		16	*Z. marina, Ulva* spp., *Sabellidae* spp.	*Z. marina* beds
AFR	France	Lagoon	G	Donation	5–10	n/a	n/a	n/a	Channel of sand with *Z. marina* beds on sides
TPO	Portugal	Lagoon	G	Donation, collection dive	3–4	2–4	19	*Z. marina*	*Z. marina* beds on sand
PPO	Portugal	Estuary	G	Collection dive	1–3	< 1	19	Artificial	Ropes and other artificial structures on heavy silt
RPO	Portugal	Lagoon	H, G	Collection dive	1–6	1–7	20	Sand, *Z. marina*, *C. nodosa*, Artificial	Sparse *Z. marina*, sand urchins and macro-algae, tunicates and artificial
MSP	Spain	Coastal	H, G	Collection dive	6–8	2–5	19	*Z. marina*	Mixed sparse seagrass beds
TFR	France	Lagoon	G	Collection dive	2–4	1–4	21	Various	Mixed and complex
GFR	France	Coastal	H	Collection dive	4.5–6	< 1	15	On benthos	Heavy silt and tunicates
GMA	Malta	Coastal	H	Collection dive	9–20	15–40	18	*Z. marina*	Sand/seagrass bed, + 70 m deep wall @ 20 m
KGR	Greece	Coastal	H, G	Collection dive transect dive	5–19	15–20	24	*Z. marina*	Mixed seagrass on slope
CGR	Greece	Coastal	G	Collection dive transect dive	2–5	15–20	26	Stones	Sponge, rock and pebble wells
VBU	Bulgaria	Coastal	G	Collection dive transect dive	5–6.5	1	25	In mixed algae, *Dictyopteris* and *Chaetomorpha* spp.	*Ulva* spp.
LCN	Spain	Coastal	H	Collection dive transect dive	6–21	10–30	21	Artificial substrates	Rock, rope and ship wreck
SEN	Senegal	n/a	H	Trade	n/a	n/a	n/a	n/a	n/a

(b) Published data								
Site	Country	Type	Species	Sampling method	Depth range (m)	Holdfast	Habitat	References
DUK	UK	Coastal	G	Survey dive	1–3	*Z. marina*	*Z. marina*	Garrick-Maidment et al. (2010)
OUK	UK	Lagoon	H, G	Interview	0–17	Algae and seagrass (G) n/a (H)	Algae (H + G), sand (H + G), mixed seagrass (G), Oyster bed (H + G)	N. Garrick-Maidment Pers. Corr.
AFR	France	Lagoon	H, G	Interview	3–20	n/a	*Z. marina*, *Z nolti*, sand, shells	Grima (2011)
GSP	Spain	Coastal	H, G	Survey dive	2.5–8	Macroalgae (G) Seagrass (H)	Sand (G), seagrass macroalgae	Valladares et al. (2011), (2013)
APO	Portugal	Estuary	H, G	Net	n/a	n/a	n/a	Veiga et al. (2009)
RPO	Portugal	Lagoon	H, G	Transect dive and net	0–7	Tunicates and shells (H + G), Artificial (G), sessile invertebrates (H + G), macroalgae (H + G), seagrass (H + G)	Mixed seagrass and macroalgae (G), sessile invertebrates (H + G), sand (H)	Curtis and Vincent (2005, 2006), Curtis (2004), Caldwell and Vincent (2012), Correia et al. (2015), Vieira et al. (2014)
RSP	Spain	Lagoon	G	Net	2–3	n/a	*C. nodosa, i*nvasive *Caulerpa*	Verdiell-Cubedo et al. (2006)
TFR	France	Lagoon	G	Transect dive	0–9	Artificial	Sand, algae and sparse seagrass	Louisy (2011)
SIT	Italy	Coastal	H	Transect dive	n/a	n/a	Sand	Canese et al. (2007)
MIT	Italy	Lagoon	H, G	Transect dive	0–5 12	Artificial (H + G)	Mixed sand, sparse seagrass, dense *Ulva* sp.	Tiralongo and Baldacconi (2014), Gristina et al. (2015)
VIT	Italy	Lagoon	H, G	Net	n/a	n/a	Seagrass (H + G), saltmarsh (G)	Franco et al. (2006)
TSL	Slovenia	Coastal	G	Transect dive	4–10	*C. nodosa*	*C. nodosa*	Bonaca and Lipej (2005)
KGR	Greece	Coastal	G	Transect dive	n/a	n/a	n/a	Issaris and Katsanevakis (2010)
MTR	Turkey	Coastal	G	Net	0–2	n/a	Seagrass, sand	Keskin (2007)
TTR	Turkey	Coastal	G	Interview	n/a	n/a	n/a	Kasapoglu and Duzgunes (2014)
NTR	Turkey	Coastal	G	Interview	n/a	n/a	n/a	Başusta et al. (2014)
WTR	Turkey	Coastal	H, G	Interview	0–30	n/a	Seagrass, rock, mud, sand	Filiz and Taskavak (2012)
ATR	Turkey	Coastal	H, G	Interview and trawl	n/a	n/a	n/a	Gurkan and Taskavak (2007)
RGR	Greece	Coastal	H, G	Trawl	12–15	n/a	seagrass	Kitsos et al. (2008)
GTU	Tunisia	Lagoons	H, G	Trammel net and dredge gear	n/a	n/a	n/a	Ben Amor et al. (2011)
GCN	Spain	Coastal	H	Transect dive	15	Macroalgae, sessile invertebrates, seagrass	Rock, sand, *C. nodosa*, macroalgae, artificial	Otero-Ferrer (2011)

### Statistical analysis

Pearson correlation was used to assess all correlation relationships between seahorse size, sex and cirri presence. Differences in seahorse length, once juvenile data (Curtis and Vincent 2005) were removed, were assessed using GLM to determine whether there was a difference between sexes and between sites (when mature seahorse *n* > 10), or a combination of the two. A post hoc Tukey pairwise comparison was used to determine at which sites seahorse lengths were significantly different. Differences in seahorse abundance were determined by Mann–Whitney test, and correlation between abundance of the two species was assessed with Pearson correlation. Deviation of sex ratios from equal was measured with Chi squared goodness of fit, and post hoc multiple test Benjamini–Hochberg correction. Correlation between abiotic parameters and species abundance was assessed with Spearman Rho. All these tests were implemented in Minitab v 17. Association of species presence with specific habitat parameters was calculated by ANOSIM in Primer v7.

## Results

### Genetic differentiation

**Fig. 2:**
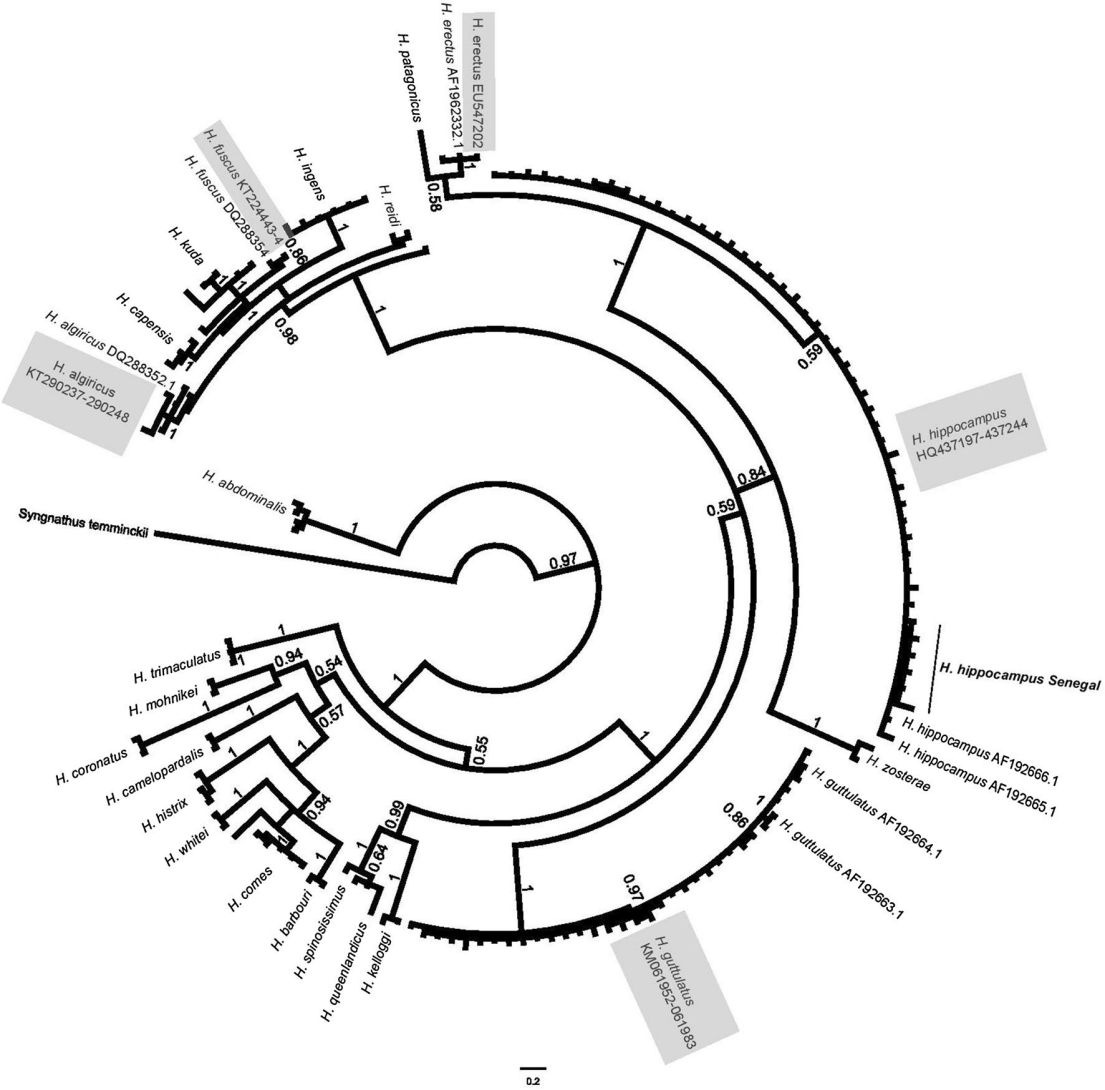
Phylogenetic tree of the relationship among *Hippocampus* species, constructed from Cytochrome b using MrBayes (GTR + γ) and shows posterior probability. Shaded labels are those generated in this study, and *H. hippocampus* from Senegal are denoted by bold text

In total, 478 seahorse specimens from 18 sites representing 10 countries (Fig. 1, Table 1) were PCR amplified and sequenced for both cytb and CR regions, with fragments trimmed to 518 and 397 bp, respectively to assist alignment. Data suggested the presence of a single specimen of *H. erectus* from the Azores (Woodall et al. 2009), three specimens of *H. fuscus* from Egypt, and five specimens of *H. algiricus* from Senegal (cytb data only shown, Fig. 2). All other specimens clearly group into two monophyletic clades corresponding to the two recognised European species *H. guttulatus* (212) or *H. hippocampus* (257). Intraspecific DNA sequence variation across all samples of *H. guttulatus* and *H. hippocampus* was low (1.23% cytb and 1.49% CR, and 1.94% cytb and 1.96% CR, respectively), and identical (*H. guttulatus*) or similar (*H. hippocampus*) to variation observed within individual populations [maximum of 1.23% for cytb (VBU) and 1.49% for CR (MSP) in *H. guttulatus*, and 1.21% for cytb (RPO) and 1.67% for CR (SEN) in *H. hippocampus*—see Woodall et al. (2011, 2015)].

### Seahorse morphology

Cirri were present on both species and the number of cirri present on seahorses varied considerably between sites and between individuals within sites. The mean cirri presence for *H. guttulatus* was 87% (40–100%, *N* = 500) across 11 sites and for *H. hippocampus* the mean was 43% (6–74%, *N* = 226) across 9 sites, covering the entire geographic range of these species. For both species the number of cirri varied more between individuals within the same site than between species; however the species differed significantly in number of cirri (Mann–Whitney, *W* = 157, *N*_1_ = 11, *N*_2_ = 98, *P* < 0.005). In *H. guttulatus,* data from this study show there is no correlation between sex and cirri presence (Pearson correlation, *R* = − 0.11, *N* = 151, *P* = 0.16), however standard length and cirri presence are significantly correlated with larger fish having cirri more often than smaller fish (Pearson correlation, *R* = 0.40, *N* = 151, *P* < 0.001). By contrast female *H. hippocampus* were more often seen with cirri than males (Pearson correlation, *R* = − 0.27, *N* = 91, *P* < 0.01).

**Fig. 3:**
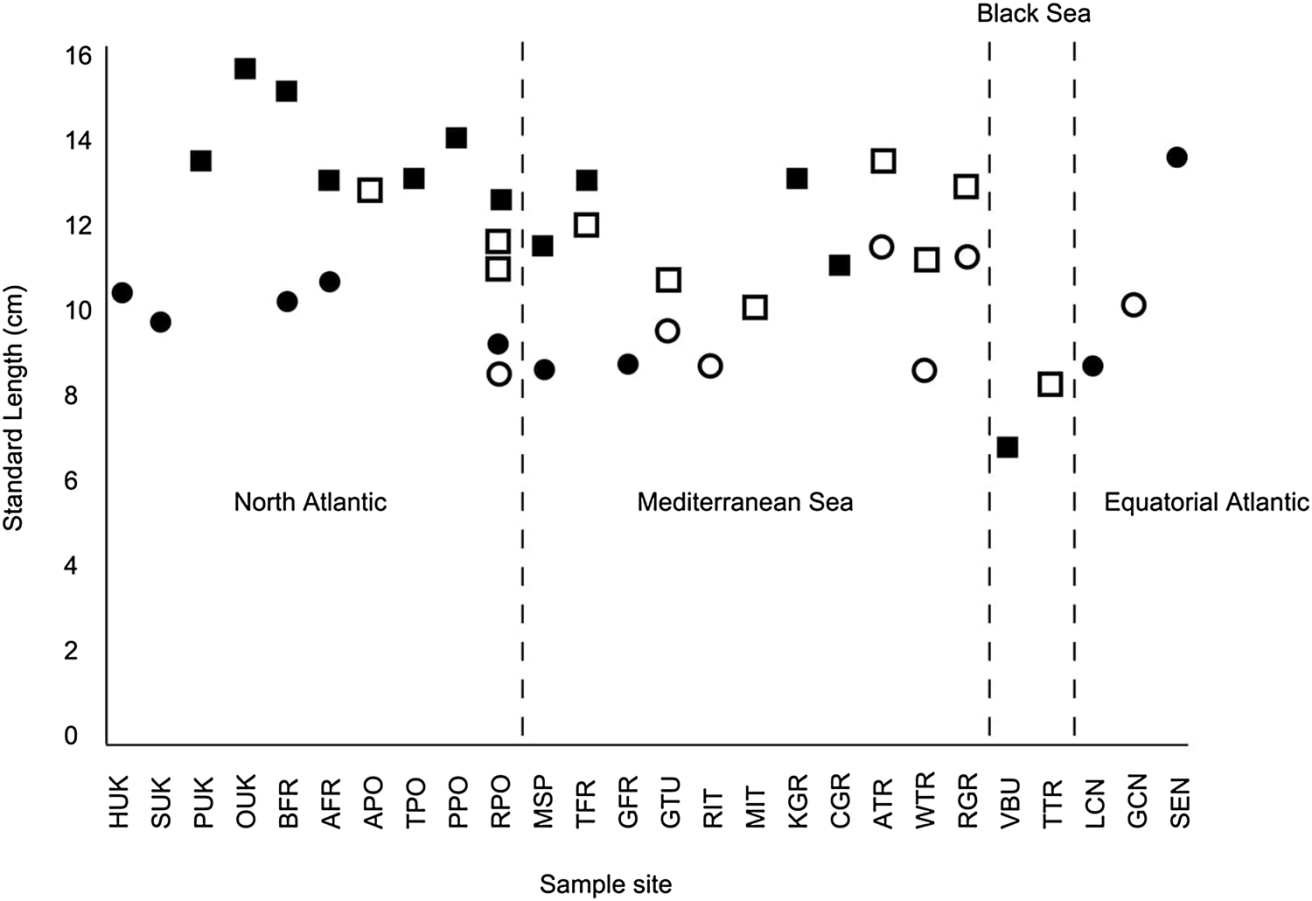
Standard length of *H. guttulatus* (squares) and *H. hippocampus* (circles) including new data and that from published studies, when *n* > 10. Filled in shapes are new data and open shapes are data from previous studies (Table 1 for site code details). Sites are grouped by region and are ordered from north to south or west to east depending on location

Standard length of *H. guttulatus* was significantly different between sexes and between sites (GLM, sex *F* = 39.4, *P* < 0.001, sites *F* = 47.7, *P* < 0.001, site and sex *F* = 0.47, *P* = 0.83). Males were significantly smaller than females, and *H. guttulatus* in the Black Sea were significantly smaller than at any other site according to Tukey’s pairwise comparisons at 95% CI. When new and published data are combined, individuals of *H. guttulatus* in the Black Sea (VBU and TTR, Fig. 1) are smaller than specimens sampled from everywhere else (Fig. 3, Table 2). In *H. hippocampus* there were significant differences in standard length between sexes, sites and the interaction of the two, accounting for 55% of the variation seen (GLM, sex *F* = 50.8, *P* < 0.001, sites *F* = 4.9 *P* < 0.005, sex and site *F* = 8.6, *P* < 0.001). Similar to *H. guttulatus,* male *H. hippocampus* were significantly smaller than the females according to Tukey’s pairwise comparisons at 95% CI, and individuals from Senegal were significantly larger than those of all other sites (Fig. 3, Table 2). In both species there is no correlation between latitude and standard length (*H. guttulatus* Pearson correlation, *R* = 0.23, *N* = 15, *P* = 0.42; *H. hippocampus R* = − 0.362, *N* = 16, *P* = 0.17).

**Table 2:** Population demographics of *Hippocampus guttulatus* (a) and *H. hippocampus* (b)

Site	Total number	Juvenile (%)	Proportion of females	*L*_s_ (cm) (min and max)	Sampling period	References
(a) *Hippocampus guttulatus*						
PUK	17	0	0.53	13.6 (8.6–18.6)	May, Aug, Oct	This study
OUK	28	n/a	n/a	15.7 (10.0–21.6)^b^	n/a	Neil Garrick-Maidment Pers. Corr.
BFR	15	1	0.60	15.2 (8.0–20.5)	June	This study
AFR	38	3	0.71	13.0 (8.6–17.4)	Sept, Oct, Nov	This study
GSP	21	14	0.33	n/a	Year-round	Valladares et al. (2011)
APO	84	n/a	n/a	12.8 (3.6–18.5)	Year-round	Veiga et al. (2009)
TPO	37	8	0.50	13.2 (9.0–20.4)	Sept, Oct	This study
PPO	42	43	0.45	13.9 (9.0–16.8)	Sept	This study
RPO	321	17	0.57	12.7 (8.7–17.9)	Sept	This study
	384	13	0.55	11.3^a^ (6.9–21.5)	May–Oct over 3 years	Curtis and Vincent (2006)
	58	10	0.57	n/a	July–Nov	Caldwell and Vincent (2012)
	1674	6	0.53	n/a	Year-round	Correia (2015)
	2042	n/a	n/a	11.7 (7.1–16.6)^b^	Year-round	Vieira et al. (2014)
MSP	19	0	0.62	11.8 (9.2–17.9)	June	This study
RSP	31	n/a	n/a	n/a (4.2–7.3)^c^	Year-round	Verdiell‐Cubedo et al. (2006)
TFR	25	0	0.36	13.0 (9.9–18.6)	June, July, Aug	This study
	114	16	0.62	12.0 (8.1–16)^b^	Year-round	Louisy (2011)
MIT	225	21	0.54	10.0 (7.0–14.0)	June–Sept	Gristina et al. (2015)
KGR	14	7	0.46	13.0 (8.0–15.7)	Sept	This study
CGR	13	0	0.53	11.2 (8.6–15.3)	Sept	This study
VBU	60	2	0.68	6.4 (4.3–9.0)	June	This study
TTR	272	n/a	0.50	8.3 (6.5–10.3)^c^	Year-round	Kasapoglu and Duzgunes (2014)
NTR	139	n/a	0.42	n/a (5.7–9.0)	n/a	Başusta et al. (2014)
WTR	135	n/a	n/a	10.8 (6.4–13.2)^cd^	n/a	Filiz and Taskavak (2012)
ATR	200	n/a	0.48	13.3 (10.0–16.5)	Year-round	Gurkan and Taskavak (2007)
RGR	279	n/a	0.54	10.8 (7.8–22.5)	Mar	Kitsos et al. (2008)
GTU	1773	n/a	n/a	12.5 (6.3–17.6)^ce^	Year-round	Ben Amor et al. (2011)
(b) *H*. *hippocampus*						
HUK	49	40	0.61	10.5 (5.6–19.8)	Sept	This study
SUK	24	33	0.50	9.9 (7.1–16.8)	April	This study
OUK	9	n/a	n/a	9.4 (5.1–15.2)	n/a	Neil Garrick-Maidment Pers. Corr.
BFR	16	0	0.50	10.2 (7.3–13.5)	June	This study
AFR	13	13	0.54	10.6 (5.7–15.7)	Sept, Oct, Nov	This study
GSP	9	n/a	0.34	n/a (11.8–17.1)	Year-round	Valladares et al. (2013)
APO	9	n/a	n/a	n/a (4.5–13.7)^c^	Year-round	Veiga et al. (2009)
PPO	6	0	0.33	8.3 (4.3–14.9)	Sept	This study
RPO	44	0	0.60	8.7 (4.3–17.6)	Sept, October	This study
	41	2	0.44	n/a (8.7–14.6)	June–Sept	Curtis and Vincent (2005)
	18	28	0.38	n/a	July–Nov	Caldwell and Vincent (2012)
	418	n/a	n/a	8.3 (5.0–13.4)^b^	Sept	Vieira et al. (2014)
	86	22	0.53	n/a	Sept	Correia (2015)
MSP	23	0	0.52	8.5 (5.1–13.4)	May, June	This study
GFR	21	0	0.61	8.5 (5.6–13.3)	July	This study
GMA	5	0	0.60	9.1 (6.4–13.4)	Aug	This study
MIT	16	6	n/a	n/a	June–Sept	Gristina et al. (2015)
RIT	46	n/a	n/a	8.4 (5.7–10.3)	March	This study
KGR	8	0	0.50	7.9 (5.7–11.1)	Sept	This study
WTR	279	n/a	n/a	8.4 (5.2–12.8)^d^	n/a	Filiz and Tasavak (2012)
ATR	29	n/a	0.27	11.3 (7.9–14.0)	Year-round	Gurkan and Taskavak (2007)
RGR	19	n/a	0.26	9.3 (6.9–10.4)	Mar	Kitsos et al. (2008)
GTU	236	n/a	n/a	10.9 (7.4–15.6)	Year-round	Ben Amor et al. (2011)
LCN	19	0	0.52	8.4 (5.6–11.9)	Nov	This study
GCN	165	20	0.58	10.2 (7.7–14.7)	Year-round	Otero-Ferrer et al. (2015a)
SEN	40	n/a	n/a	13.7 (10.8–18.1)^d^	n/a	This study

Number of seahorses samples, percentage of juveniles, sex ratio, standard length (*L*_s_) and sampling period

^a^At first reproduction

^b^Height

^c^Total length

^d^Dried specimens

^e^ID as *H. ramulosus*

### Seahorse population density

The total number of seahorses observed at each site varied considerably depending on species and survey method. The mean abundance of *H. guttulatus* was 3.15 ± 1.08 (mean ± SE) seahorses per diver hour for collection dives, and in transect surveys density was 0.076 ± 0.06 ind. m^−2^. For *H. hippocampus*, the mean abundance was 2.49 seahorses per diver hour but only one transect was conducted for this species, so no mean density is reported. When data from published studies were combined with transect dives in this study, mean abundance was greater in *H. guttulatus* than *H. hippocampus* (*H. guttulatus*: 0.04 ± 0.01 ind. m^−2^ *N* = 12; *H. hippocampus*: 0.003 ± 0.001 ind. m^−2^, *N* = 7) (Table 3).

**Table 3:** Mean population abundance include new and published data new for *H. guttulatus* and *H. hippocampus*

Site	Seahorses per diver hour		Seahorses per m^2^ of transect		References
	*H. guttulatus*	*H. hippocampus*	*H. guttulatus*	*H. hippocampus*	
BFR	1.565	1.130	–	–	This study
GSP	–	–	0.007	–	Valladares et al. (2011)
TPO	3.000	0.006	–	–	This study
PPO	6.000	0.980	–	–	This study
RPO	–	–	0.073	0.007	Curtis and Vincent (2005)
	–	–	0.004	0.001	Caldwell and Vincent (2012)
	–	–	0.107	0.005	Correia (2015)
SFR	0.980	0.001^a^	–	–	This study
	–	–	0.001–0.014^b^	–	Louisy (2011)
GFR	–	7.000	–	–	This study
GMA	–	0.190	–		This study
SIT	–	–	–	0.006	Canese et al. (2007)
MIT	–	–	0.018	> 0.001	Gristina et al. (2015)
VIT	–	–	0.001	> 0.001	Franco et al. (2006)
TSL	–	–	0–0.08	–	Bonaca and Lipej (2005)
KGR	1.070	1.000	0.020	0	This study
	–		0.004	–	Issaris and Katsanevakis (2010)
CGR	1.220	0.002	0.004	0	This study
VBU	8.240^c^	–	0.203	–	This study
LCN	–	2.100	–	0.002	This study
GCN	–	1.760/0.840^d^	–	–	Otero-Ferrer et al. (2015b)

^a^Only one seahorse seen but first report of this species here

^b^Range given not mean abundance

^c^Abundance estimate was limited by underwater genetic sampling procedures

^d^Calculated from 15 min dive transects

In all locations (*N* = 6) where the two species occurred sympatrically, the density of *H. guttulatus* was greater than that of *H. hippocampus* and in all but one case this was by at least an order of magnitude greater (Table 3). In the Ria Formosa, Portugal, where observations covered many years, *H. guttulatus* was always found in greater abundance than *H. hippocampus* when the Ria Formosa was considered as one site, but in some locations within the Ria Formosa only one of the two species was found (e.g. Curtis and Vincent 2005).

There is no significant difference in abundance of *H. guttulatus or H. hippocampus* between sites where it cohabits or not with its congener (*H. guttulatus* new data Mann–Whitney, *W* = 18, *N* = 10, *P* = 0.8; *H. hippocampus* all data Mann–Whitney *U* = 12, *N* = 7, *P* = 1). There was no correlation between the density of the two seahorse species for either the data collected as ind. diver hour^−1^ (Spearman’s rho, *r*_s_ = − 0.1, *N* = 5, *P* = 0.8) or in the Ria Formosa, Portugal, when abundance was measured as ind. m^−2^ (Spearman’s rho, *r*_s_ = 0.2, *N* = 3, *P* = 0.8).

### Population structure

Each seahorse population had its own unique combination of characteristics, regards juvenile percentage and sex ratio (Table 2). In both species, the proportion of observed juveniles varied widely, from 0 to 43% for *H. guttulatus* and from 0 to 40% for *H. hippocampus*. However, on average, this was about 17% across both species, and there appeared to be no effect of time of year. No *H. guttulatus* populations were significantly male-biased, but TFR2 and VBU were both significantly female-biased (Chi Squared, TFR2: *χ* ^2^ 6.9, *P* < 0.01; VBU: *χ* ^2^ 8.1, *P* < 0.01), following Benjamini–Hochberg multiple comparison correction (false recovery rate 0.1). No *H. hippocampus* populations had a significant sex bias.

### Abiotic parameters of seahorse habitat

There was a large variation in the environmental parameters of locations where seahorses were found. When all survey methods were considered, seahorses were found at depths ranging from 1 to 55 m, but when only dive surveys were included, seahorses were just found at 1–21 m. Most *H. guttulatus* (86%) were found at 2–5 m depth, from surveyed depths of 1–28 m. By contrast, just 19% of *H. hippocampus* specimens were found in 2–5 m depth and at two sites (GMA&LCN) all were found much deeper (≥ 20 m). Neither water temperature nor visibility correlate with *H. guttulatus* abundance (Spearman’s rho, water temperature *r*_s_ − 0.03, *N* = 8, *P* = 0.96, visibility *r*_s_ − 0.50, *N* = 8, *P* = 0.20), or with *H. hippocampus* abundance (Spearman’s rho, water temperature *r*_s_ − 0.38, *N* = 6, *P* = 0.34, visibility *r*_s_ 0.04, *N* = 6, *P* = 0.94) when density is calculated as ind. diver hour^−1^.

### Seahorse habitat and holdfast preference

**Fig. 4:**
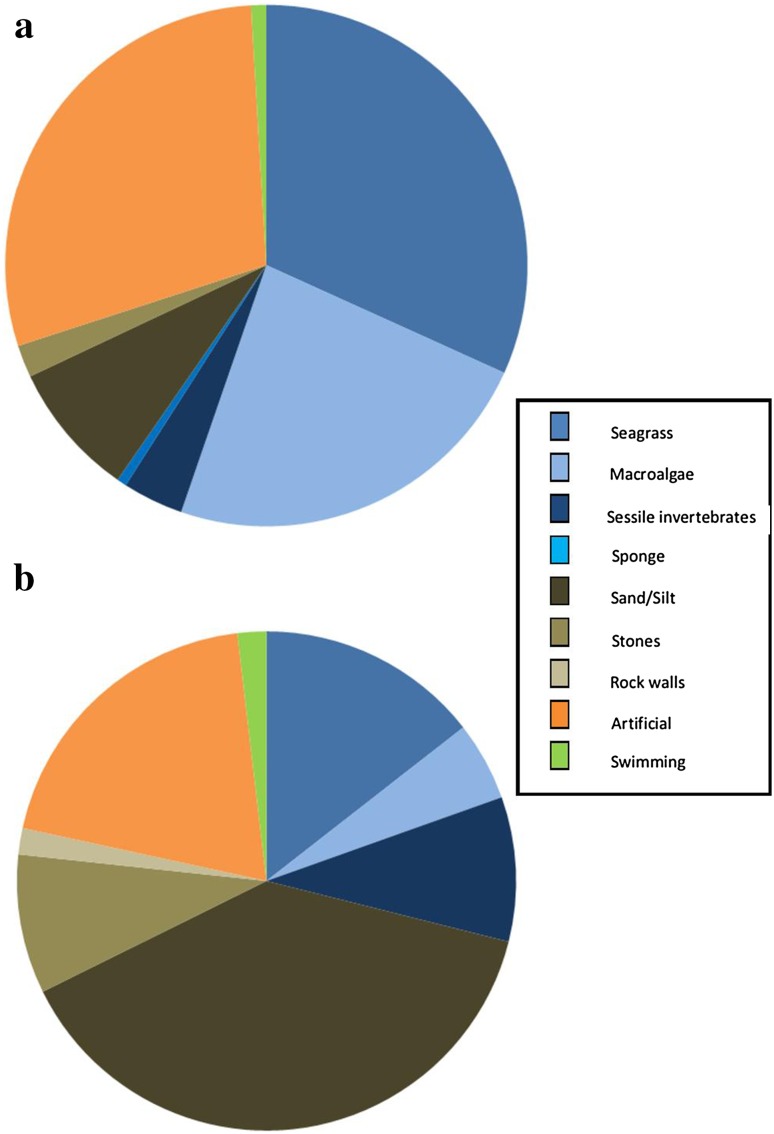
Holdfast substrate types utilized by *H. guttulatus* (**a**) and *H. hippocampus* (**b**). Blues Organic, Browns Inorganic, Orange Artificial, Green No holdfast (swimming)

New data from this study show most seahorses encountered used holdfasts, with only 1% of *H. guttulatus* and 2% of *H. hippocampus* seen while they were actively swimming. The seagrass *Zostera marina* was present at most sites (67%) where *H. guttulatus* was observed. However seagrass beds were not the dominant habitat (6.1 SE ± 4.2%) in any of the sites assessed with transect dives, although it was the most popular holdfast, accounting for just under half of *H. guttulatus* holdfasts (Fig. 4) (Table 1a). The second most popular holdfast type was artificial structures (> 25% of seahorses). Artificial holdfasts were items such as tyres, fishing gear, ropes, bricks and pier supports. Transect dives revealed that most seahorses were found in complex habitats on a sand/silt substrate. These habitats included seagrass beds, sessile invertebrates, algal species and artificial structures (Table 4).

**Table 4:** Survey dive data, habitat types observed from transects and quadrat surveys

Site	Species	Transect habitat		Quadrat habitat and percentage cover					
		Dominant habitat	Other habitats	Bottom type	% cover	Flora/Fauna	% cover	Artificial	% cover
KGR	*H. hippocampus* *H. guttulatus*	Sand	*Zostera/Cymodocea* mixed beds Sea urchins	Sand Gravel Stones Rock	41.2 38.0 4.1 0.2	*Zostera* *Cymodocea* Urchins	11.3 3.0 0.2	Brick	2.0
CGR	*H. guttulatus*	Sand/Rock covered in algae	Gravel, urchin, sea cucumber, gravel, sponge	Sand Stones Rock	51.0 33.6 7.4	*Zostera* Urchins Anemone Sponge	4.0 0.8 0.8 1.6	Brick	0.8
VBU	*H. guttulatus*	Sand	Algae	Sand	96.0	Mixed *Chaetomorpha* and *Cladophora* spp. Hermit crab	3.0 1.0		
LCN	*H. hippocampus*	Sand	Rock, rope, tyre	Sand	98.0	Rock	0.5	Rope tyre	1.0 0.5

Most sites that *H. hippocampus* inhabited were mixed habitats of open sand or silt. These substrata often had added complexity due to the presence of sessile invertebrates, artificial structures or macroalgae. In addition, at six sites at least one species of seagrass was observed in the general vicinity of the seahorses. *Hippocampus hippocampus* was most commonly found settled into depressions in the sediment. However artificial structures were the most common holdfast used when they were present (19%) (Fig. 4).

Detailed descriptions of seahorse habitat and holdfast preference are lacking from many previous studies. However when reported, seagrass was present, but not dominant, at most of the sites (86%). The seagrass observed was a mix of genera: *Cymodocea*, *Zostera* and *Posidonia*. However, when holdfast preference was recorded, macroalgae and artificial substrates were more often used by *H. guttulatus*, and invertebrates used by *H. hippocampus*. Data were transformed into presence/absence due to the variety of methods and detail reported, but no individual factors could account for seahorse presence (ANOSIM, seagrass *R* = 0.26, macroalgae *R* = − 0.01, sand *R* = 0.09, substrate complexity *R* = 0.18 and artificial objects *R* = − 0.06).

## Discussion

The present study provides the first comprehensive review of genetic and demographic information on European seahorses throughout their geographic range. It also provides data on variability in morphology and habitat use between and within *H. guttulatus* and *H. hippocampus*. Despite sampling constraints, these baseline data provide useful information for conservation assessments for these data poor species of conservation concern (Woodall 2012a, b).

### How many species of seahorse are there in European waters?

Genetic data from 478 samples representing 18 locations collected in 10 countries revealed the presence of five species of seahorse in NE Atlantic, Mediterranean and Black Sea waters: *H. algiricus*, *H. erectus*, *H. fuscus*, *H. hippocampus,* and *H. guttulatus*. One specimen from the Azores was identified as *H. erectus* using genetic techniques, and subsequent morphological examination has confirmed this was the first observed occurrence of this species in the eastern Atlantic Ocean (Woodall et al. 2009). Another species, *H. fuscus*, is an example of Lessepsian migration and was identified from specimens originating from northern Egypt, although congruence of morphological characteristics was not possible as tissue was donated rather than whole specimens. This species had previously been recorded in the south–eastern Mediterranean Sea (Golani and Fine 2002; Gokoglu et al. 2004) and extant populations have been observed as far north as Turkey (Gokoglu et al. 2004). *Hippocampus algiricus*, native to North–West African coasts (Czembor 2012), was observed in the Canary Islands (Otero-Ferrer et al. 2015b). By far the two most frequently seen species are *H. guttulatus* and *H. hippocampus*, which are native European species (Lourie et al. 2016).

There was no genetic evidence within the samples tested for the presence of cryptic species or of substantial within-species differentiation (i.e. of sub-species level). This conclusion could be drawn as all clades included reference sequences from known species and displayed no geographically based or morphology-related structuring, despite the high degree of intra-specific morphological variation observed within and among sites (Otero-Ferrer et al. 2017). The only substantial within-species genetic differentiation observed was that associated with haplotypes of *H. hippocampus* from Senegal and some *H. guttulatus* haplotypes from the Black Sea (Fig. 2). However in both cases no genetic and morphological correlation can be assumed, as both Black Sea and Senegalese populations also possessed the widely distributed common haplotypes of their respective species (Woodall et al. 2011, 2015). Levels of intra-specific genetic variation in cytochrome b sequences for both *H. guttulatus* and *H. hippocampus* (1.23 and 1.94%, respectively) were similar to values reported for other seahorse species such as *H. barbouri* (1.72%, Lourie et al. 2005) and *H. erectus* (1.44%, Boehm et al. 2013). Intra-specific variation in the Control Region in *H. guttulatus* and *H. hippocampus* (1.49 and 1.96%, respectively) likewise was within values given for other species such as *H. abdominalis* (2.23%, Nickel and Cursons 2012), *H. capensis* (1.49%, Teske et al. 2003) and *H. ingens* (2.10%, Saarman et al. 2010).

The lowest inter-specific genetic variation among seahorses has been reported between *H. reidi* and *H. algiricus*, consistent with these species having the most recent common ancestor (Teske et al. 2007a), whereas most other species pairwise comparisons show much greater genetic divergence (e.g. 5.75% between *H. erectus* and *H. patagonicus,* Boehm et al. 2013). Although not conclusive, such levels of inter-specific and intra-specific genetic variation across seahorse species suggest that the intra-specific variation observed across the entire geographical ranges of *H. guttulatus* and *H. hippocampus* is consistent with these comprising single undifferentiated species, and is also congruent with the limited morphological difference seen. This is an important conclusion, as previous studies based solely on morphological data proposed new subspecies and species in the Mediterranean Sea (Kuiter 2009) and Black Sea (Lourie et al. 1999b), that have subsequently been synonymised on the basis of the genetic data (Woodall 2012a, b). This information is crucial for surveying, assessing, monitoring, and managing the two focal species of this study, but could have wider ramifications for seahorse taxonomy globally, where it is common for morphological characters alone to be used to describe species. Integrated taxonomy is recommended for many species (Schlick-Steiner et al. 2010; Chen et al. 2011) and conclusions from this study suggest this is particularly important for seahorses where morphological differentiation can be challenging.

### Morphology in *H. hippocampus* and *H. guttulatus* is not consistent across their range or within populations

Previous studies have relied on a subset of morphological characters that are distinctive in seahorses (Lourie et al. 1999b) to determine taxonomy. Data on two commonly used characteristics (presence/absence of cirri, standard length) show a differing proportion of individuals with cirri in each population of both species, and results indicate that the presence and number of cirri are unreliable characters for European seahorse species identification. This result is congruent with the few other studies that recorded this morphological character (Curtis and Vincent 2006; Curtis 2006; Louisy 2011; Tiralongo and Baldacconi 2014; Otero-Ferrer et al. 2015a). Cirri presence was more likely on larger *H. guttulatus* supporting findings by Curtis (2006), however this is not always the case (Garrick-Maidment pers. comm.), suggesting that the conditions required to exhibit this character are highly complex. In *H. hippocampus* however, females were more likely to have cirri. This was congruent with a study in the Canary Islands by Otero-Ferrer et al. (2015a) which reported females were more likely to have cirri than males. Tagged *H. hippocampus* can shed cirri over time (JMR Curtis, unpublished data) and in some locations in the UK, *H. hippocampus* are never seen with cirri (Garrick-Maidment, unpublished data).The general shape of cirri on *H. guttulatus* and *H. hippocampus* is often different, with cirri branching in a different manner (for example images see Figure S1). However these differences are often unclear unless specimens are compared simultaneously. As the genetic data clearly confirm the presence of just two native European species, this current study can therefore confidently confirm cirri presence or absence is not a consistent or diagnosing feature within species, concurring with Curtis (2006).

Sexual dimorphism, in the form of a shorter standard length of males, was observed in *H. guttulatus* and *H. hippocampus*. This was previously reported for *H. guttulatus* (Curtis and Vincent 2006) and in many other seahorse species (Foster and Vincent 2004), but the current study is the first to indicate that this is consistent for European seahorses across their entire geographic range. Sexual dimorphism is generally a characteristic associated with polygamous species, rather than monogamous ones like seahorses (Emlen and Oring 1977; Jones and Avise 2001), although *H. guttulatus* is serially monogamous across breeding seasons (Naud et al. 2008). In seahorses the mating system is thought to be result from morphology, behaviour of mate competition, and the energy required to produce eggs and brood them (Kvarnemo and Simmons 2013).

Adult *H. guttulatus* from the Black Sea were significantly smaller than those from all other locations. This was observed for both new data (VBU) and published data (TTR—Kasapoglu and Duzgunes 2014). In addition, *H. hippocampus* from Senegal were larger than those from other sites. A significant size difference of seahorses from different populations has not been observed previously, however a large range of sizes have been reported for both *H. hippocampus* and *H. guttulatus* (Table 2), morphological variation has been seen across Macaronesia and W. Africa in *H. hippocampus* (Otero-Ferrer et al. 2017), and phenotypic plasticity is recognised in other seahorse species (Teske et al. 2007b). In some species of pipefish, which are in the same family as seahorses, lengths are significantly different between populations (e.g. *Syngnathus floridae*, Mobley and Jones 2009; *Syngnathus typhle*, Rispoli and Wilson 2008), and another pipefish species, *Syngnathus abaster*, appears to be morphologically divergent across different locations (Cakic et al. 2002; Veiga et al. 2009; Ben Alaya et al. 2011). Based on mtDNA data, these studies suggest the morphological differences between populations are probably linked to genetic differentiation in *S. abaster*, whereas ecological factors are a more likely cause for the morphological variation observed in other pipefish species as no genetic correlation is seen (Mobley and Jones 2009). In the present study the size of both focal species was different across sites. Additional studies are required to elucidate which location-specific factors correlate with the observed size differences in seahorses. Despite the apparent trend for seahorses to be larger in the most northerly locations (a proxy of seasonal variation in temperatures), these findings are not significant and therefore neither European seahorse species follow Bergmann’s rule. This rule states larger individuals are found in colder environments, and smaller ones in warmer ones. This nonconformity could be a sampling artefact, but may also reflect that an organism’s size is influenced by a complex range of ecological and evolutionary processes (Berke et al. 2013), and seahorse survival requirements are known to be complex. It is therefore unsurprising that they show morphological variation across their geographic range as an adaptation to different local conditions, similar to that observed in their confamilial *Syngnathus leptorhynchus* (Wilson 2009).

### Population demographics

Seahorse density was generally low, but patchy and highly variable. New abundance estimates were within values given in other studies of both species (Table 1), but few report density using ind. diver hour^−1^, which comprises the majority of new data in this study. No direct comparison between ind. m^−2^ and ind. diver hour^−1^ was possible. Safety considerations during diving, such as depth, water clarity, current flow rate and boat traffic limited the possible search area within known seahorse sites (Curtis and Vincent 2005; Curtis et al. 2017). All new study sites were chosen because seahorses had previously been observed at them, therefore abundance presented is artificially inflated. The choice of search method has also been shown to influence abundance recorded, which in most cases will have also inflated abundance reported (Correia et al. 2016). However, mean density for *H. hippocampus* from new data presented here are within values extrapolated from previous studies of *H. hippocampus* (Otero-Ferrer et al. 2015a). Data from both species combined (Goffredo et al. 2004) suggests that this method could be useful for surveys and comparisons with distance transect measures, should be a priority. Densities reported in previous studies are from transects or focal grids. The latter are often chosen to encompass areas of high seahorse density (e.g. Bell et al. 2003) and therefore seahorse densities from focal studies would be artificially higher compared to randomly placed transects. The seahorse densities given per area surveyed in the present study were generally similar to those previously reported in these species (e.g. Gristina et al. 2015), but greater than those reported for other seahorse species (Foster and Vincent 2004). This may be an artefact of the sampling protocol as mentioned above, a species-specific characteristic or peculiarity of sample location. Both abundance measures (ind. diver hour^−1^, ind. m^−2^) employed in the current study showed that across their range *H. guttulatus* abundance was greater than that of *H. hippocampus*. The abundance of *H. guttulatus* was always greater than that of *H. hippocampus* in locations where they co-occurred. Other studies have shown this pattern over limited geographic areas (Curtis and Vincent 2005; Caldwell and Vincent 2012; Gristina et al. 2015). Just one other study reports percentage abundance of co-occurring seahorse species, which revealed the same composition of species and the same most abundant species (Murugan et al. 2008). Counter to this in pipefish the species of greatest abundance appears to be related to season (Ripley and Foran 2006) and microhabitat preference (Malavasi et al. 2007).

The female-biased sex ratio of *H. guttulatus* in two of the sites, in the Black Sea and southern France (VBU in this study and TFR2 in Louisy 2011) is unexpected as serial monogamy reported for *H. guttulatus* in an ex situ trial and over 2 years in the wild (Naud et al. 2008) and over 4 years in the wild at a UK site (Garrick Maidment unpublished data) predicts an equal sex ratio. An independent study (new data in the present study) of site TFR reported an equal sex ratio, which might suggest the female bias of the Louisy (2011) may be an anomaly and additional data should be collected to investigate this further. Seasonal changes in the sex ratio have been reported for *H. zostera* (Strawn 1958), a female biased population was documented in *H. abdominalis* (Martin-Smith and Vincent 2005), and an equal sex ratio was observed for *H. comes* (Perante 2002), suggesting a variety of sex ratios can be observed across seahorse species. All *H. hippocampus* populations in the present study had an equal sex ratio, with most individuals found as male/female pairs (pers. obs.), although seasonal changes have been indicated in one study (Otero-Ferrer et al. 2015a). This interesting difference between species should be investigated further to determine if this phenomenon is a possible characteristic for niche partitioning in these species, especially as mating behaviour studies have not yet been conducted for *H. hippocampus*.

The number of juveniles seen in surveyed populations is fewer than adults. However, juveniles could be observed less frequently that adults due to the sampling method and regime or as a result of an ontogenetic habitat shift. Most studies of other seahorse species (reviewed in Foster and Vincent 2004), including *H. guttulatus* (Correia 2015; Gristina et al. 2017), also documented low proportions juveniles, however a high proportion has been found in some populations of *H. capensis* (Lockyear et al. 2006). There is a precedent for ontogeny in seahorses (*H. comes*, Morgan and Vincent 2007; *H. whitei*, Harasti et al. 2014). Further research is required to understand this aspect of behaviour in European seahorses, although has been observed in *H. hippocampus* in the Canary Islands (Otero-Ferrer et al. 2015a). This is especially important as best practice dictates that the effective management and conservation of species needs to address all life stages (Gerber and Heppell 2004).

### Can seahorse population location be predicted by environmental parameters?

In this study, no individual environmental parameters could define the presence of seahorses, species abundance, or determine which species was present. However survey locations were not picked at random, with only locations where seahorses were already known to be present being studied, which may have limited our ability to detect environmental parameters that are unsuitable for seahorses. In order to model where seahorses may occur, it is important to identify how different locations and habitats fulfil the needs of seahorses. These factors could include environmental parameters under extreme events like storms and extended periods of heat (Cohen et al. 2017). Correlation of *H. guttulatus* abundance and temperature has been reported for multiple populations within the Ria Formosa (Correia 2015), although neither visibility nor temperature appeared to correlate with seahorse sightings in a UK site (The Seahorse Trust 2014).

When only new data were analysed, *H. guttulatus* was most commonly seen in complex habitats and *H. hippocampus* in simpler ones; supporting previous location-specific studies (Curtis and Vincent 2005; Canese et al. 2007; Correia 2015; Garrick-Maidment 2011; Gristina et al. 2015; Otero-Ferrer et al. 2015a). Niche partitioning was also observed in sympatric pipefish (Kendrick and Hyndes 2003; Malavasi et al. 2007 and in the pygmy seahorses *H. denise* and *H. bargibanti* Smith et al. 2012). There is inconclusive evidence of the importance of *Zostera marina* as a required or preferred habitat for *H. guttulatus,* as although it did not always co-occur with seahorse populations, when present it was most often used by *H. guttulatus* as a holdfast. Although *Z. marina* itself could be important for *H. guttulatus,* it is more likely to be the food availability as infauna and epifauna associated with the seagrass (Bostrom and Bonsdorff 1997) that is driving habitat preference, as is the case with pipefish (Ryer and Orth 1987). Individuals of both *H. guttulatus* and *H. hippocampus* were observed using artificial objects as holdfasts. The use of artificial holdfasts is seen in many seahorse species (Rosa et al. 2007; Clynick 2008; Faleiro et al. 2008), and could be an important factor in management measures as they could provide refuge for seahorse prey items or function as seahorse aggregation devices (Correia et al. 2013, 2015), but this apparent behavioural preference may be an observer artefact because they are more easily seen on this type of object.

The apparent wide range of habitats means that predicting the likelihood of these species’ presence from habitat and environment parameters alone is challenging. This is an important consideration for environmental assessments that are made before potentially damaging activities (e.g. coastal construction). The requirements of such assessments differ across states, however habitat is often used as a precursor to determine which species (such as seahorses) could be at risk. Our findings suggest that this strategy would not be suitable for determining potential impacts on *H. hippocampus* and *H. guttulatus*. Furthermore, as seahorse conservation efforts are currently associated with seagrass conservation (e.g. Heritage Lottery Fund 2014), much of the variation in European seahorse habitat may be missed if seagrass beds alone are conserved, despite this habitat being important for many other species (McCloskey and Unsworth 2015).

### Important new insights and future research suggestions to enable appropriate conservation measures

This study provides the first synthesis of data on habitat, population demography, morphology and genetics of the two native European seahorse species *H. guttulatus* and *H. hippocampus* from across their geographic range. We report the large variety of habitats in which these fish are found, failed to identify one simple parameter that predicts the presence or abundance of these seahorses, but note that seagrass is not always associated with either species. Data show that the morphology of specimens should be carefully considered together with genetic data, in an integrated approach, in order to assign species identifications (Feulner et al. 2007); such accurate integrated identification is vital in order to allow international legal mechanisms and international agreements such as the Convention on International Trade in Endangered Species (CITES 2015) to work effectively.

Emerging techniques such as eDNA screening could be applied to locate hitherto unknown populations, which would be valuable for understanding the distribution and ecology of these species. The differences in abundance observed between the two species suggest different conditions are required for these species to thrive, but these exact parameters are yet to be determined. Niche partitioning is expected in congeneric species, and further observations to determine any differences in prey items (Kitsos et al. 2008), morphology and behaviour would be an interesting contribution to determine how management measures differentially impact the two species.

As both *H. guttulatus* and *H. hippocampus* are currently classified as Data Deficient (IUCN 2015), any range-wide conservation measures should also encompass long-term monitoring so that the threat status of these iconic fish can be reassessed. Especially as population trends are unknown in many locations. Applying the precautionary principal, widespread threats from coastal development and non-target fisheries, combined with the large geographic range, low genetic differentiation (Woodall et al. 2011, 2015) and small adult home ranges (Curtis and Vincent 2006; Curtis et al. 2017) of European seahorses suggests that a network of protected areas would be part of an effective scheme for seahorse in situ conservation. These protected areas should combine different habitat types and safeguard shallow waters, should be large enough to account for changing environmental conditions and be close enough to each other to ensure genetic and demographic connectivity. Although unlikely to be the panacea for seahorse conservation due to wider ranging issues such as climate change, where they are fished or habitat is directly damaged by human activities, protected areas have been shown to increase the size of the seahorses within them (Yasue et al. 2012) resulting in increased brood size, and thus increased resilience of the population.

## Electronic supplementary material

Below is the link to the electronic supplementary material.

Supplementary material 1 (PDF 366 kb)

Supplementary material 2 (DOCX 12 kb)
