# Supplementary material for: A synthesis of European seahorse taxonomy, population structure, and habitat use as a basis for assessment, monitoring and conservation
Source: Mar Biol. 2017 Dec 5;165(1):19. doi: 10.1007/s00227-017-3274-y (PMC5717113; doi:10.1007/s00227-017-3274-y)
Supplement: Supplementary file 1 — Supplementary material 1 (PDF 366 kb) [file 227_2017_3274_MOESM1_ESM.pdf]

Figure S1: Photos to illustrate the morphological variation across species and the distinguishing morphological characteristics

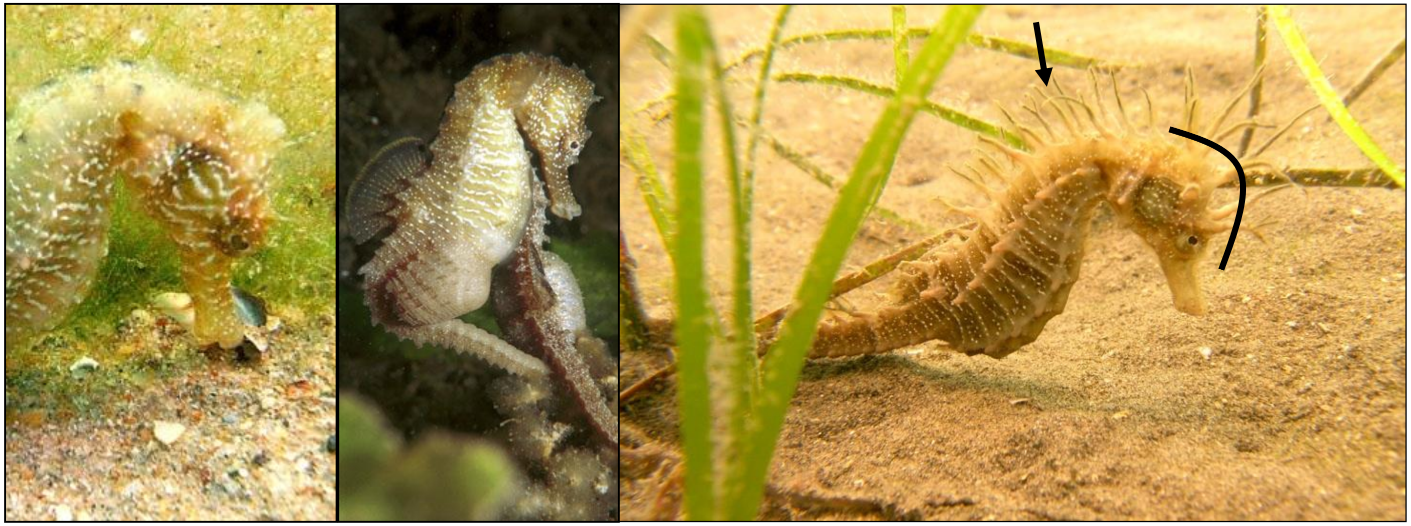

Bulgaria | Southern France | Southern Spain

Hippocampus guttulatus

Note the curved head shape and when present thick cirri with few branches

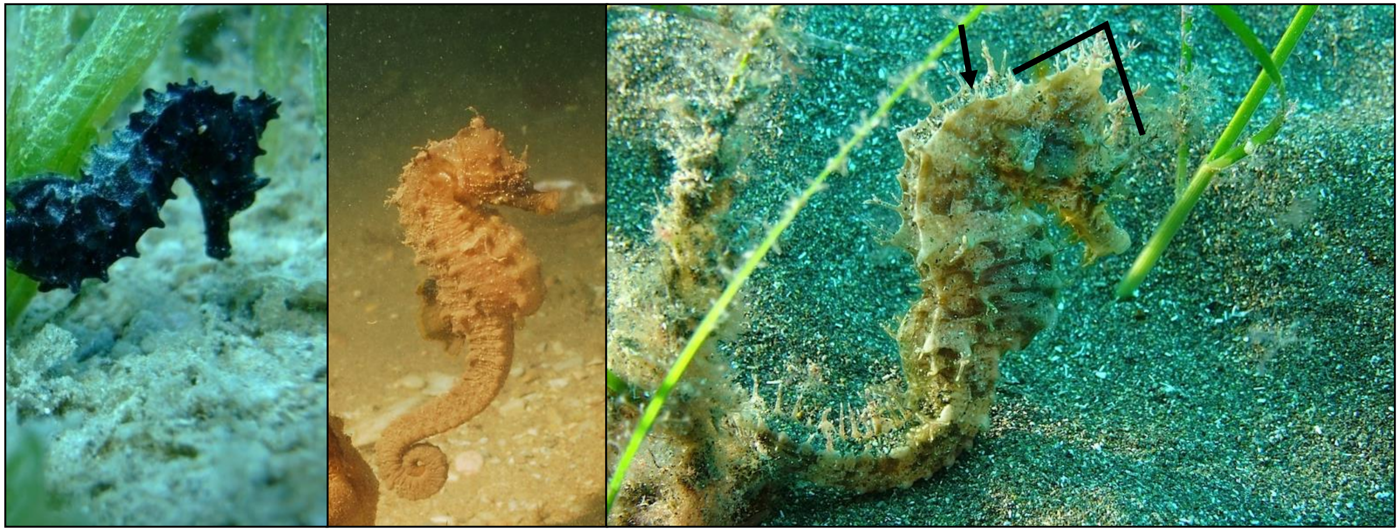

Greece | United Kingdom | Canary Islands

Hippocampus hippocampus

Note the triangular head shape and when present thin cirri with many branches
