# Supplementary material for: A synthesis of European seahorse taxonomy, population structure, and habitat use as a basis for assessment, monitoring and conservation
Source: Mar Biol. 2017 Dec 5;165(1):19. doi: 10.1007/s00227-017-3274-y (PMC5717113; doi:10.1007/s00227-017-3274-y)
Supplement: Supplementary file 2 — Supplementary material 2 (DOCX 12 kb) [file 227_2017_3274_MOESM2_ESM.docx]

Table S1: Genbank accession number for DNA sequences used in the study

| Species | Control region | Cytochrome B |
| --- | --- | --- |
| *H. guttulatus* | KM062016-KM061984 | KM061952-KM061983 |
| *H. hippocampus* | HQ437280-HQ437245 | HQ437244-HQ437197 |
| *H. erectus* | EU547205 | EU547202 |
| *H. fuscus* | KT224445-KT224447 | KT224443-KT224444 |
| *H. algiricus* | KT290239-KT290243 | KT290237-KT290238 |
